# Patient journey with Charcot-Marie-Tooth Disease – A German patient survey study

**DOI:** 10.1186/s13023-026-04236-2

**Published:** 2026-02-03

**Authors:** Helena F. Pernice, Susann May, Felix Mühlensiepen, Sebastian Spethmann, Ricarda Kneitz, Agata Mossakowski, Katrin Hahn

**Affiliations:** 1 Department of Neurology, Charité University Medicine, Berlin, Germany; 2 Department of Cardiology, Angiology and Intensive Care Medicine, Deutsches Herzzentrum der Charité (DHZC), Campus Charité Mitte, Charitéplatz 1, 10117 Berlin, Germany; 3 Charité – Universitätsmedizin Berlin, Corporate Member of Freie Universität Berlin, Humboldt Universität zu Berlin, Campus Charité Mitte, Charitéplatz 1, 10117 Berlin, Germany; 4 DZHK (German Centre for Cardiovascular Research), Partner Site Berlin, Berlin, Germany; 5 Berlin Institute of Health at Charité – Universitätsmedizin Berlin, Charitéplatz 1, 10117 Berlin, Germany

**Keywords:** Patient journey, CMT, Hereditary neuropathy, Healthcare, Survey

## Abstract

**Background:**

Charcot-Marie-Tooth (CMT) and related disorders represent one of the largest groups of inherited neurological disorders. Long considered incurable, the first disease-modifying treatments are currently being evaluated in clinical trials. However, frequent misdiagnosis or delayed recognition are common, hindering timely treatment necessary to prevent permanent disability. In this study, we conducted a survey among patients from our CMT clinic and the German patient advocate group to capture patients’ perspective on the diagnostic journey and to explore solutions for earlier diagnosis and improved access to specialized care.

**Results:**

270 CMT patients participated in the survey. The average time between symptom onset and first physician contact was 5.2 years with a symptom to diagnosis time of 13.7 years. Patients who were first seen by an orthopedic specialist had a longer diagnostic delay compared to those seen by a neurologist or geneticist. Diagnostic hospitalizations occurred in 46% of cases. More than half of the patients were initially misdiagnosed, which prolonged the diagnostic journey and impaired the patients’ general health.

**Conclusions:**

Our study highlights that patients with CMT in Germany regularly face a prolonged diagnostic odyssey before receiving a correct diagnosis. Lengthy diagnostic pathways involving frequent doctor visits and hospital stays, as well as misdiagnosis, lead to inappropriate treatment, and high emotional and economic burden. Late diagnosis may limit the effectiveness of upcoming therapies that rely on early treatment initiation. New strategies need to be defined to decrease the costs for both patients and healthcare systems and prepare for upcoming therapies.

**Supplementary Information:**

The online version contains supplementary material available at 10.1186/s13023-026-04236-2.

## Background

Inherited neuropathy or Charcot-Marie-Tooth disease (CMT) is a rare disease causing slowly progressive distal weakness and sensory loss and affects 1 in 2500 individuals [1]. Patients often present in childhood or adolescence with symptoms such as weak performance in school sports, running difficulties, and foot deformities such as high arched feet. Although traditionally considered incurable, symptomatic treatment including foot surgery [2], orthotic management [3] and physiotherapy or occupational therapy [4], has important implications on patients’ prognosis and disability. Furthermore, promising new disease-modifying or gene-targeted therapies are in preparation for clinical trials, heralding a new era for treatment in CMT [5]. CMT care is usually provided in outpatient settings by general neurologists, with inpatient treatment mainly required for surgical treatments and rehabilitation. Being a rare disease, specialized healthcare professionals are rarely available, although finding knowledgable clinicians is a main concern of patients [6]. Many clinicians are inexperienced with signs and symptoms of CMT, leading to misdiagnosis and lack of care [7]. This imposes a considerable economic burden on the healthcare system, particularly through indirect costs resulting from progressive disability and inadequate management [8]. However, timely diagnosis and continuous access to care is essential for any treatment in neuromuscular disease such as CMT, causal or not, to prevent permanent disability [9, 10]. While there several studies exist on specific features such as the burden of late diagnosis in the patient care path and diagnostic journey in rare disease in general [11–13], in specific neuromuscular disease [14–19], or single reports of patient journeys in CMT [7, 11, 12], no systematic quantitative study on the patient perspective on diagnosis and care in CMT has been performed to our knowledge. To close this gap, we investigated the diagnostic journey and care for patients with CMT in Germany. In doing so, we aim to provide a foundation for tackling the most urgent care deficits, and explore the usability of new technologies.

## Methods

### Patient survey design and distribution

**Fig. 1:**
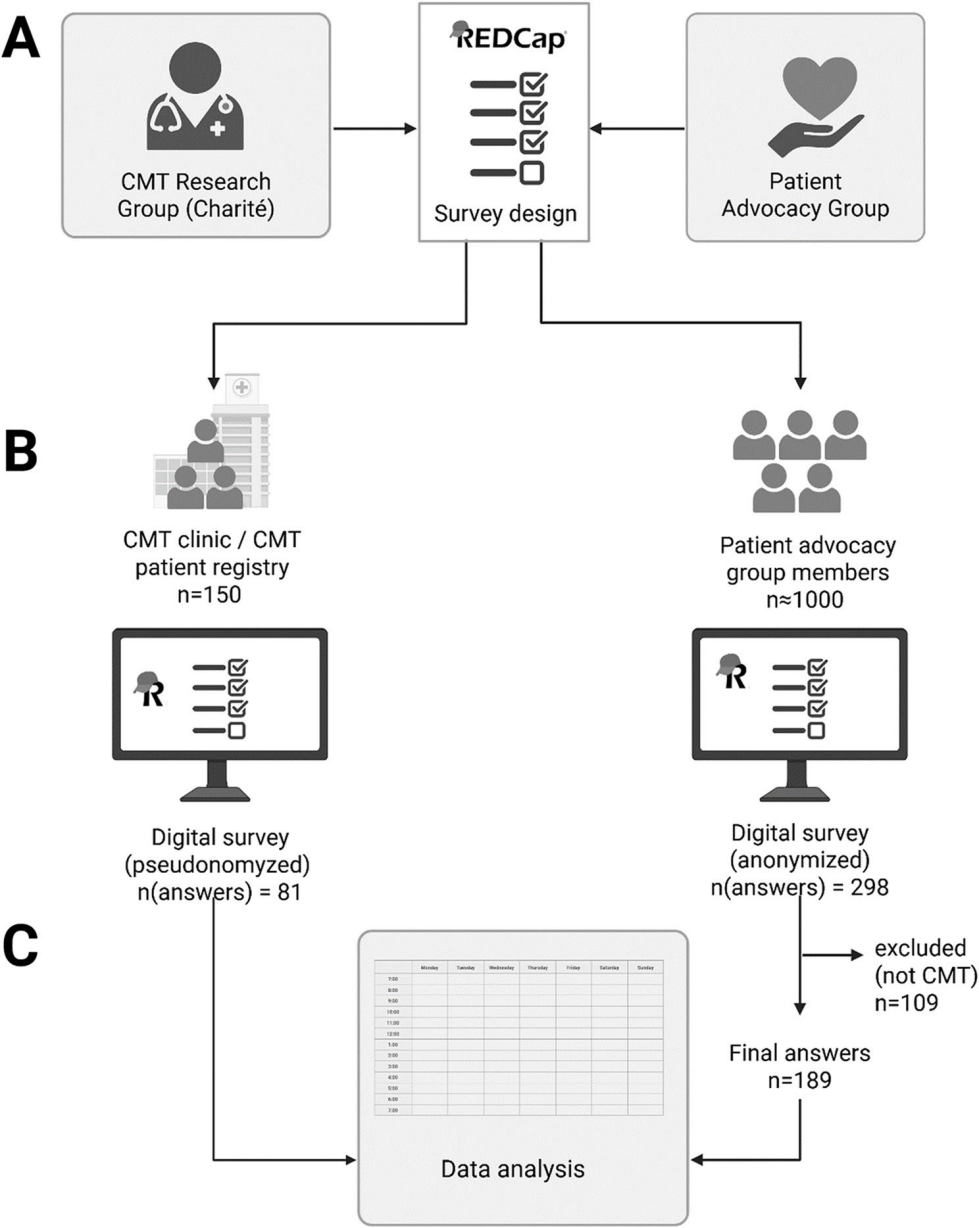
Protocol of patient survey. (**A**) Survey design using the REDCap platform by the CMT research group at Charité and patient advocate representatives. (**B**) Survey distribution via the CMT clinic and registry as well as the patient advocacy group. (**C**) Only patients with CMT were included and used for data analysis. CMT= Charcot-Marie-Tooth disease. Figure created with biorender.com

This cross-sectional patient survey study was conducted with the aim to better understand the patients’ perspective of the diagnostic journey and care in inherited neuromuscular disease, specifically CMT. Questionnaires were designed using the REDCap electronic data capture tool hosted at Charité – Universitätsmedizin Berlin, Germany [20, 21], in collaboration between clinicians specializing in CMT care and patient advocate representatives (Fig. 1, A). The target group of the questionnaire were patients with genetic neuromuscular disease of all ages, specifically CMT. Surveys contained 23 questions (German language) organized into the following sections: (1) General data, including diagnosis, sex, current age, education, ethnical origin, and current health state, (2) clinical contacts before the diagnosis with questions regarding symptom onset, first clinical or specialist contacts, as well as number of clinical visits the physicians’ specialities, (3) diagnosis, asking for burden of diagnostics, misdiagnosis and their consequences, age of diagnosis and family history, as well as communication of the diagnosis, (4) follow-up care, such as frequency of clinical visits, source of information on the disease and specialized care, as well as opinions regarding digital care strategies. The survey ended with an open text field for additional comments. The full survey (translated to English language) is available as Supplemental Table 1. The survey was pilot-tested with 10 patients to identify necessary changes in terms of formatting and wording. Minor revisions were made accordingly. Final surveys were distributed between October 1st 2024 and April 1st 2025 via the CMT clinic at Charité – Universitätsmedizin Berlin as well as via the German patient advocacy group for neuromuscular disease (Deutsche Gesellschaft für Muskelkranke GmbH, DGM) federal association of the state Berlin and CMT patient group (collectively around 800 members) as depicted in Fig. 1, B. All surveys were completed digitally (no paper versions were provided) and were completed by patients themselves or their relatives. Participants under the age of 18 completed the surveys unter supervision or in companion of legal guardians, which was a requirement in the survey instructions. There was no compensation for survey completion. To prevent multiple responses, surveys were distributed in a targeted manner. Survey anonymity and confidentiality was provided via the REDCap anonymous survey tool. Access was restricted to the research group of the CMT Registry Study. The study conforms with the principles outlined in the Declaration of Helsinki, the Good Clinical Practice guidelines, the Geneva Declaration, the Belmont Report, and the laws and regulations applicable in Germany. The institutional review board of the Charité – Universitätsmedizin Berlin approved the study (EA2/169/22). The Consensus-Based Checklist for Reporting of Survey Studies (CROSS) [22] was used.

### Data analysis and statistics

Inclusion criteria required a confirmed diagnosis of CMT and study consent (Fig. 1, C). In the anonymous survey, patients were only included if the diagnosis “CMT” or “HSMN” for hereditary sensory motor neuropathy, or a specific subtype (e.g., “CMT1A”) was stated, patients with other or less clear diagnosis (e.g. “peripheral neuropathy”) were excluded. Patients were selected using single-stage sampling. We used descriptive statistics to describe demographic variables. Missing data is depicted in respective analyses, non-response error was addressed as potential bias. Normal statistical distribution was assessed by visualization by histograms. When normally distributed, data is depicted by means ± standard deviation, when not normally distributed as median and range. Comparative analysis between groups of two was performed using the Mann-Whitney-U test and between groups of three using the Kruskal-Wallis test. Statistics were performed using GraphPad Prism 10.5.0.

## Results

### Patients and general demographics

270 German patients (57.4% female) between 8 and 90 years (mean 55 years) responded to the survey between October 2024 and April 2025 (response rate ≈ 30% [289/~1000]). Out of these, 81 patients were treated at the specialized CMT clinic at Charité – Universitätsmedizin Berlin, Berlin, Germany. Specific subtypes were available for 67 patients (representing 25% of all patients and *n* = 52, 65%, out of the CMT registry group, and *n* = 15, 8%, out of the anonymous group). Most frequent subtypes are listed in Table 1.

Most patients had vocational training (apprenticeship) or higher education (129 patients representing 47%, 36 patients representing 13% had completed university). Symptom onset ranged from 0 to 77 years, with a median of 23.7 years (also see Suppl. Figure 1, A). Phenotypic information at the age of onset could only be elaborated for participants from the CMT Registry study (see Suppl. Figure 1, B). More than 2/3 of patients reported a moderate (47.4%) to bad or very bad (22.4%) state of health (see Table 1) at the time of the survey.

**Table 1:** Basic demographics and characteristics of patients who answered the survey (*N* = 270)

	*N or mean*	% or range	*N (answers)*
Number of patients	270	100	270
Diagnosis CMT	267	98.9	270
CMT1A	13	19.4	67
CMT1B	4	6.0	67
CMTX	9	13.4	67
SORD	4	6.0	67
HNPP	8	11.9	67
other	29	43.3	67
Median age of disease onset (first symptoms)	17	0–74	266
Age at the time of answering the questionnaire in years	55	8–90	270
< 30	26	9.6	270
30–40	30	11.1	270
40–50	37	13.7	270
50–60	70	25.9	270
60–70	66	24.4	270
> 70	45	16.7	270
Female sex	156	57.8	270
Education			
University	36	13.3	270
Qualified training or apprenticeship	129	47.8	270
School diploma	24	8.9	270
Subjective health state at the time of answering the questionnaire			
Very good	25	9.3	270
Good	59	21.9	270
Moderate	128	47.4	270
Bad	49	18.1	270
Very bad	9	3.3	270

### From first clinical contact to diagnosis

**Fig. 2:**
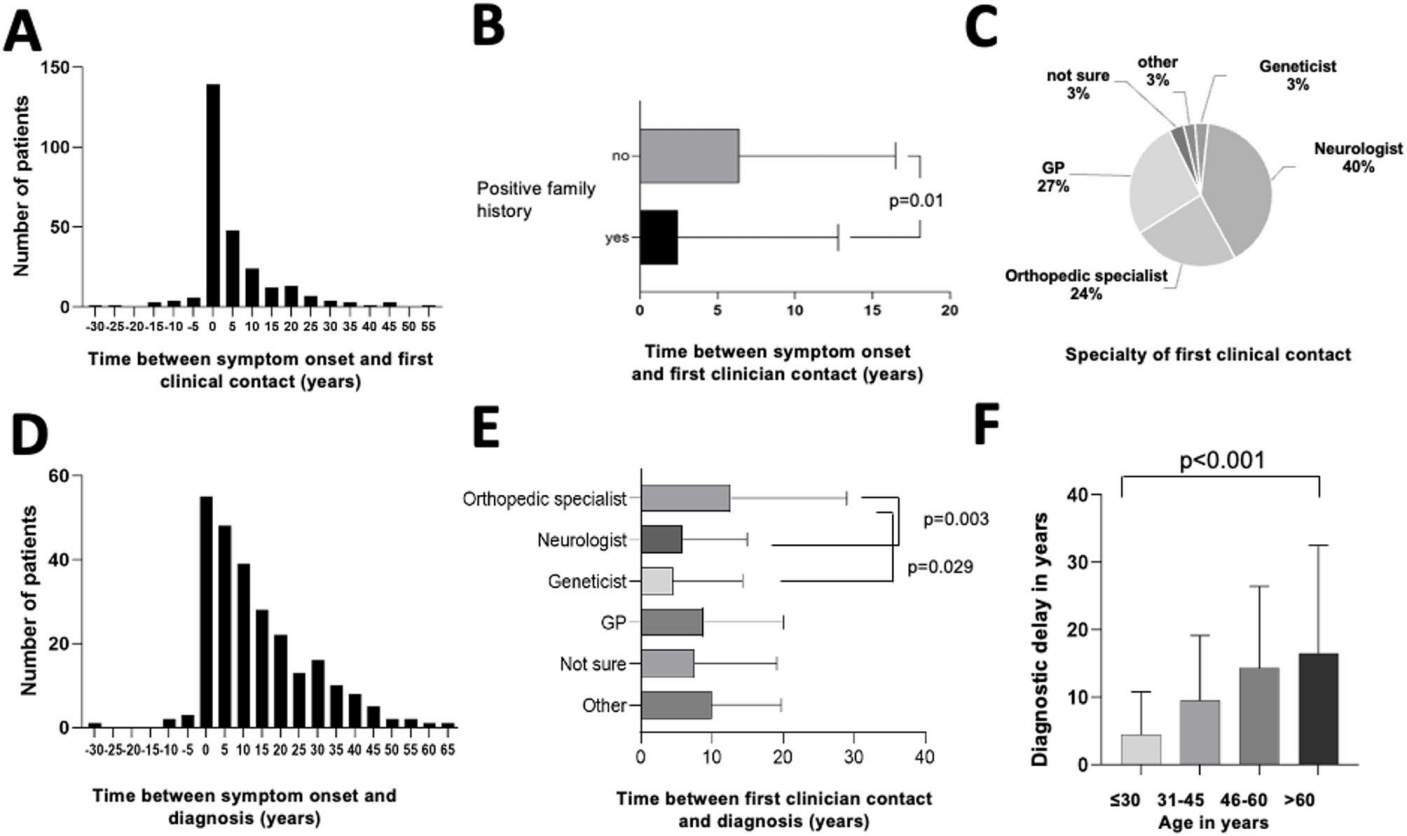
Diagnostic journey from symptom onset to diagnosis, depicting (**A**) histogram of delay between symptom onset and first clinical contact, (**B**) bar plot of delay between symptom onset and first clinical contact of participants with and without positive family history, (**C**) pie chart of specialists first contacted upon symptom onset, (**D**) histogram of delay between symptom onset and diagnosis, (**E**) bar chart of diagnostic delay depending on physician that first saw the patient, and (**F**) bar chart of diagnostic delay depending on age at time of answering the survey

Patients first consulted a physician on average 5.2 years after symptom onset (range: 30 years before to 53 years after, see Fig. 2, A). 63.6% of patients who saw a physician before symptom onset (14/22) reported to have a positive family history. Patients who knew the disease from family members also saw physicians significantly earlier than patients with no positive family history (*p* = 0.01; Mann-Whitney-U test, see Fig. 2, B); potentially due to suspicion of disease even before recognition of first symptoms. While the biggest group participants (40%) first presented to a neurologist regarding their symptoms, almost a quarter of participants saw an orthopeadic sugrean first, and a relevant percentage (27%) first contacted their GP because of their symptoms (Fig. 2, C).

The average time between symptom onset and correct diagnosis was 13.7 years ranging between 30 years before symptom onset and 66 years after symptom onset (Fig. 2, D). Being a genetic disease, 9 out of 256 (3.5%) patients assumed their diagnosis from positive family history before symptom onset. Whether this was due to predictive testing or because of family members’ symptoms was not specified in the survey. The time between the first contact with a physician and the correct diagnosis was 8.3 years, ranging from 5 years before symptom onset to 65 years of living with the disease. Interestingly, patients whose first clinical contact was a neurologist (median delay = 0 years; range=-4-40 years; *n* = 107; *p* = 0.0028) or geneticist (median delay = 0 years; range = 0–28 years; *n* = 8, *p* = 0.029) were diagnosed significantly earlier than patients seen by an orthopedic specialist (median delay = 4 years; range= − 5–65 years; *n* = 64, Fig. 2, E). There was no difference in diagnostic delay (time between first symptom and diagnosis) in patients first seen by a GP or pediatrician (median delay = 4 years; range=-1-52 years; *n* = 62) compared to neurologists or geneticists (Fig. 2, E).

There was no difference between sexes in diagnostic delay (mean[women] = 10 years; range=-30-66 years; mean[men] = 10 years; range=-12-60 years, *p* = 0.583; Mann-Whitney-U test, data not shown). Interestingly, diagnostic delay significantly differed between the generations, showing higher delay in patients from older generations (mean diagnostic delay [“Generation Z” or younger, born after 1995] = 4.4 years; mean diagnostic delay [“Generation Y”, born 1981–1995] = 9.5 years; mean diagnostic delay [“Baby boomer”, born before 1965] = 16.5 years; mean diagnostic delay [“Generation X”, born 1965–1980] = 14.3 years, *p* < 0.001, Kurskal-Wallis test, Fig. 2, F).

### Patient distress during diagnostic journey and communication of diagnosis

**Fig. 3:**
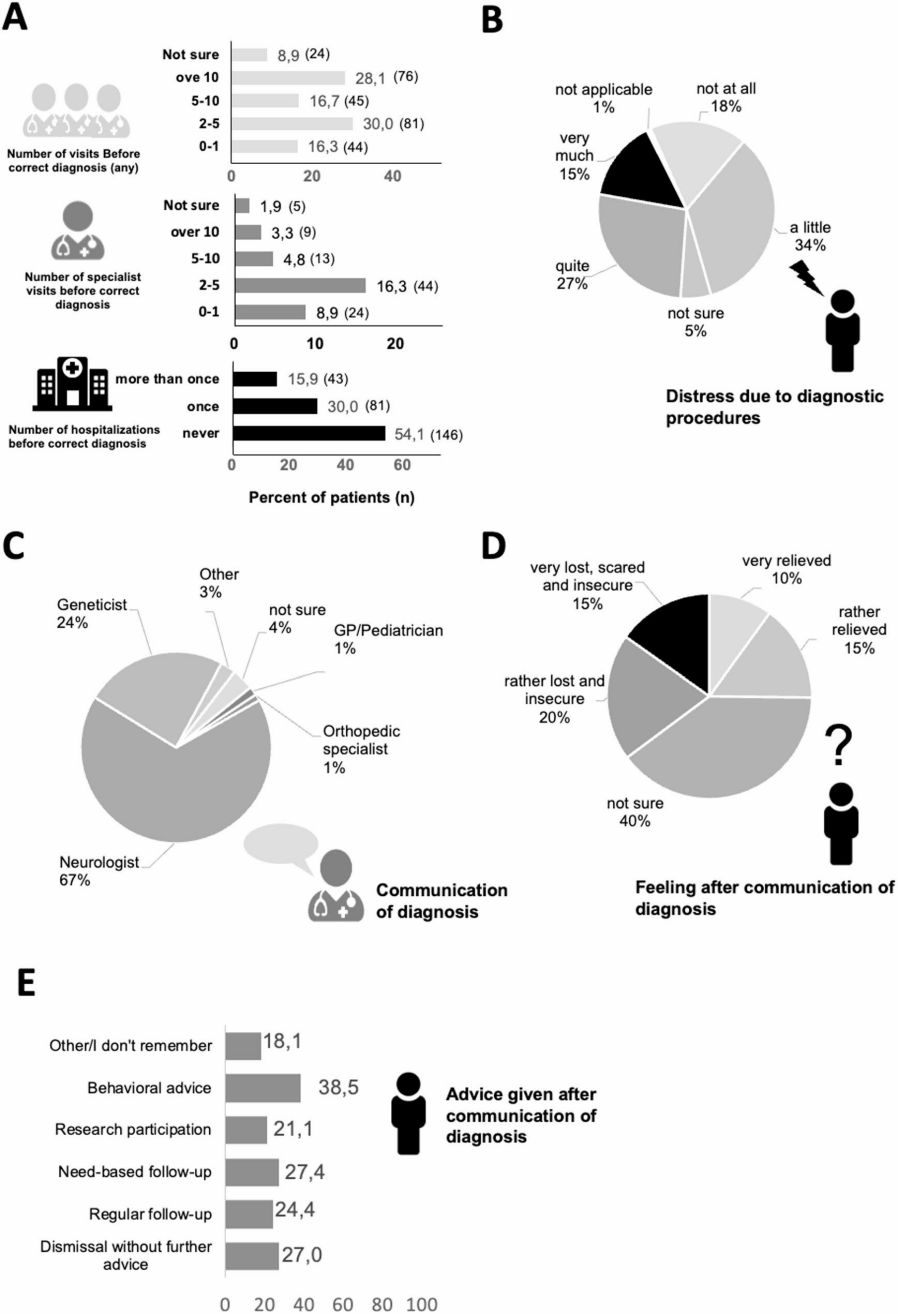
Patient distress in diagnostic journey and communication of diagnosis. (**A**) Bar charts depicting the number of visits to any physician (top), specialists (middle), and hospitalizations (bottom) because of CMT symptoms before diagnosis, (**B**) pie chart of perception of distress because of diagnostic procedures, (**C**) pie chart of specialty of physician communicating the diagnosis, and (**D**) pie chart of patient distress after hearing the diagnosis, and (**E**) bar chart depicting advice given after communication of the diagnosis

To assess the diagnostic journey, we asked how often patients saw clinical healthcare professionals before receiving the final diagnosis (Fig. 3, A). Almost one third of patients (28.1%, *n* = 76) reported more than 10 visits to any physician before receiving a correct diagnosis, 16.7% (*n* = 45) had 5–10 visits, and another 30.0% (*n* = 81) had 2–5 visits. Regarding hospitalizations, 30.0% (*n* = 81) of patients were admitted for diagnostic purposes, with 15.9% (*n* = 43) hospitalized more than once (Fig. 3, A). Although genetic testing can be considered the most straightforward way to confirm CMT, complex neurological workup often preceded genetic testing including invasive procedures such as lumbar punctures, electrophysiological studies, and MRIs, which were often perceived as very (15%, *n* = 40) or quite (27%, *n* = 72) stressful, bothersome or painful (Fig. 3, B). 18% perceived diagnostic procedures as unproblematic (*n* = 48).

In 67% of cases (*n* = 181), neurologists communicated the final diagnosis, in 24.1% geneticists, smaller numbers were represented by general practitioners or pediatricians (*n* = 4; 1.5%), orthopedic surgeons (*n* = 3; 1.1%), or other physicians (*n* = 7; 2.6%) (Fig. 3, C). After receiving the diagnosis, 35.2% of patients felt uncertain or very lost (*n* = 54 and 42, respectively), while 25.2% of patients felt relieved or rather relieved (*n* = 27 and 41, respectively, Fig. 3, D). Here, 10% of patients (*n* = 27) were dismissed because of lack of therapy, 39.6% (*n* = 107) were told to return only for new complaints, and 15% (*n* = 41) were offered follow-up visits. 20.0% (*n* = 54) were offered study participation, 15.2% (*n* = 41) were offered behavioral advice, and 10% (*n* = 27) reported that they did not remember or were unsure (Fig. 3, E).

### Misdiagnosis and implications

**Fig. 4:**
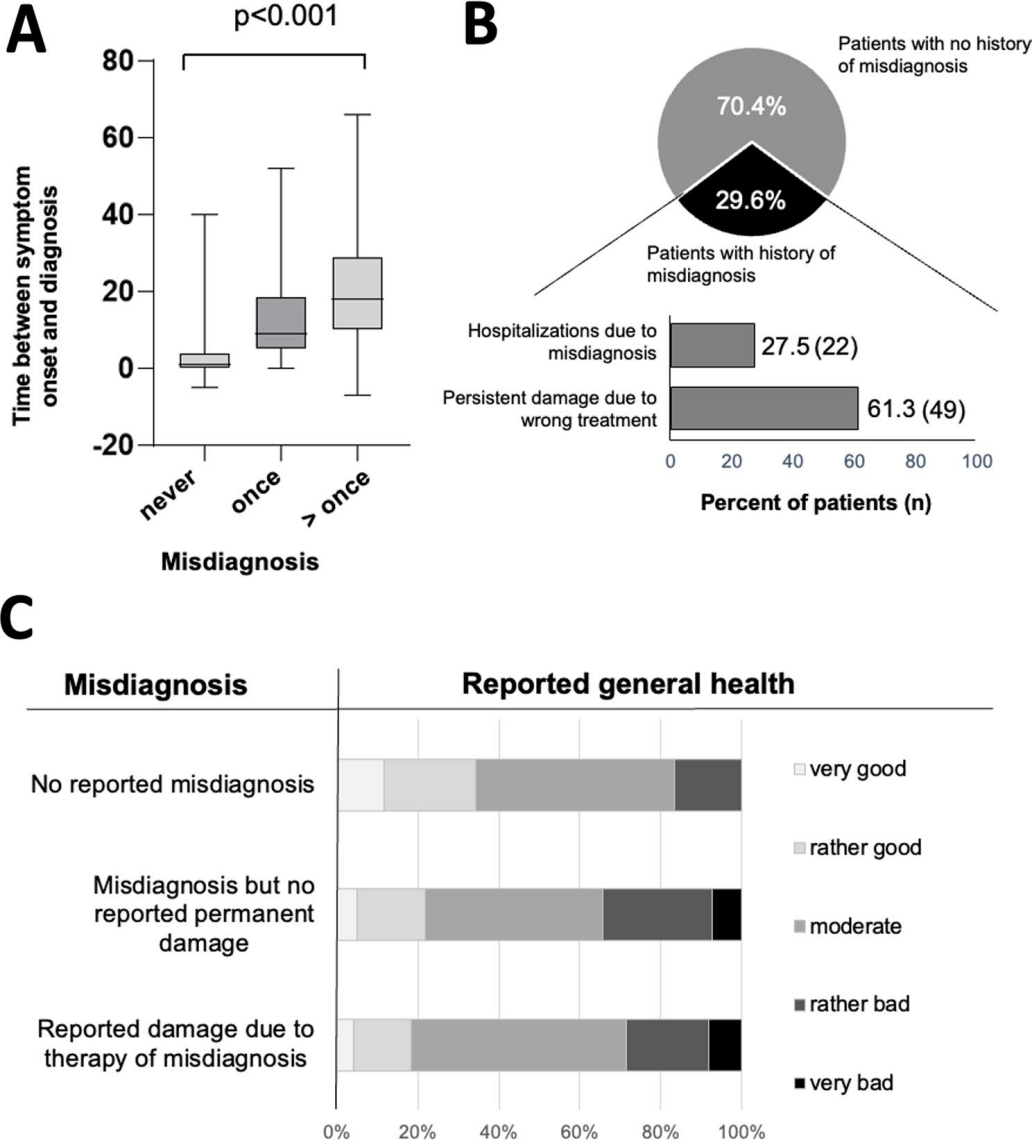
Impact of misdiagnosis on (**A**) diagnostic delay depicted in boxplot, (**B**) bar chart showing amount of patients with misdiagnosis and consequent hospitalizations or damage due to wrong treatment, and (**C**) bar chart of reported general health depicted in likert scale. Statistics: Kruskall-Wallis test

51.9% (*n* = 140) of the patients received a wrong diagnosis at least once. Those patients also reported significantly longer diagnostic journeys with a median diagnostic delay ranging from 1 year in the group with no misdiagnosis to 9 and 18 years in those with one or more misdiagnoses, respectively (Fig. 4A, *p* < 0.001). We also asked whether patients thought they had received wrong therapies because of misdiagnoses (Fig. 4, B). 29.6% (*n* = 80) patients confirmed this, out of which 61.3% (*n* = 49) of patients felt that their wrong therapy had caused permanent damage to their health, and 27.5% (*n* = 22) reported to have been hospitalized because of a past misdiagnosis (Fig. 4, B), accounting for 37.2% (16 out of 43) of patients that reported hospitalizations. Patients who reported health damage from prior misdiagnoses also reported significantly worse general health at the time of the survey compared to those without a history of misdiagnosis (*p* = 0.008, see Fig. 4, C).

### Quality of current care

**Fig. 5:**
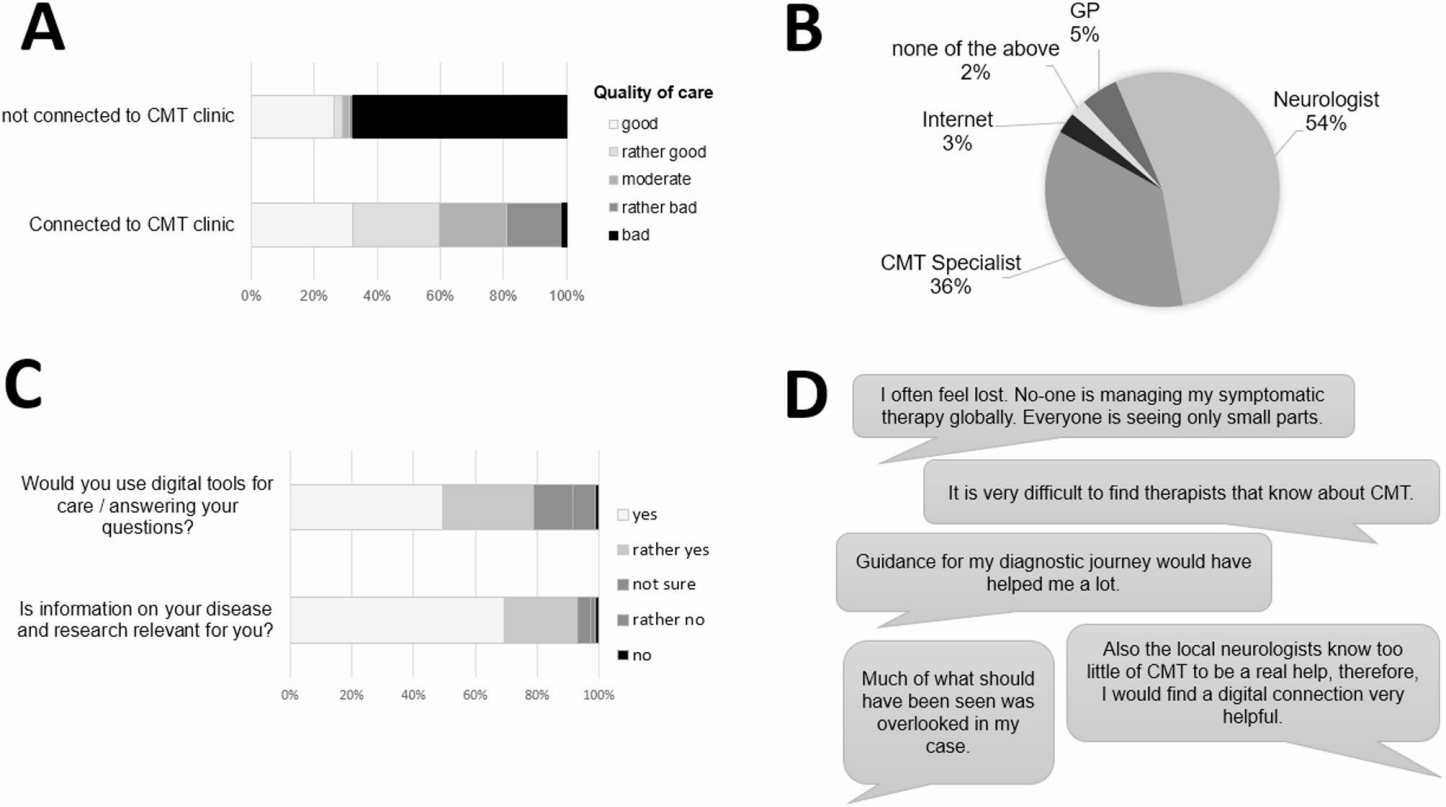
Current care evaluation and patient opinions on needs for improvement. (**A**) Likert scale regarding patient evaluation of current care quality depending on connection to a CMT clinic. (**B**) Pie chart of who patients ask when they have questions regarding their disease. (**C**) Importance of disease information and openness towards digital care tools. (**D**) Selected patient statements on the current care and patient journey

Generally, the majority of patients reported good or rather good care (58.5%; *n* = 158, and 25.9%; *n* = 70, respectively). 84.6% reported receiving symptomatic care such as physiotherapy and/or aids such as orthotics. 82.1% of patients were in touch with a specialist center at least once (Fig. 5). Importantly, Patients treated at a CMT specialist center reported significantly better care than those not treated at such a center (*p* < 0.001, Mann-Whitney-U test, Fig. 5, A), but 32.7% of patients had a delay of more than 10 years between their first visit to any physician and contact to a specialist center (see Suppl. Figure 1, C).

We also tried to evaluate the need for clinician contact post-diagnosis. Most patients reported visiting a physician because of CMT symptoms once or twice a year (46.2%), and 30.7% of patients more than twice a year. Patients said that when they had questions regarding their disease, they most often asked their neurologist or CMT specialist (53.7% and 35.9%, respectively), 8% generally looked up their questions on the internet, and 5.2% of the patients asked their GP or pediatrician (Fig. 5, B).

### Patient perspectives on key problems and digital solutions

To develop solutions for the substantial variability and prolonged diagnostic journeys reported in the survey, we also asked patients about their overall care experiences, importance of up-to-date information, and openness towards digital care (Fig. 5, C and D). The majority (93.0%) expressed a high or rather high need for reliable information on their disease or ongoing research, and 78.9% stated they would (probably) use digital tools such as apps or telemedicine. Interestingly, this openness did not differ between patients younger than 60 years and those 60 years and older (*p* = 0.238, Mann-Whitney-U test). Further needs stated by patients are depicted in Fig. 5, D, underlining the relevance of availability of specialized care.

## Discussion

In summary, this retrospective patient survey showed that patients with CMT in Germany experience significant diagnostic delays, largely due to frequent misdiagnoses, complex diagnostic journeys, and delays in accessing specialized CMT care.

Long diagnostic journeys impose substantial psychological and financial burdens. Previous studies analyzing the psychological impact of delayed diagnosis in rare disease showed that the longer the diagnostic journey the higher the need for psychological support [23]. In contrast to other studies of rare disease diagnosis, we observed no difference in the diagnostic delay between men and women [23]. The diagnostic delay in patients with CMT was similar to other neuromuscular disorders, such as Fabry disease (21 years), Gaucher disease (20 years) [19], longer than in hereditary amyloidosis (about 3.5 years) [24], and significantly longer than in usually non-hereditary neuromuscular diseases such as Amyotrophic lateral sclerosis (1 year) [16] and myositis (2 years) [15]. This suggests that especially inherited neuromuscular disorders share a fundamental lack of disease detection, potentially due to the significantly slower progression compared to myositis and ALS, which are typically acquired diseases and only rarely inherited in case of ALS. This underscores the need for increased awareness, as well as new diagnostic tools or biomarkers to better detect slowly progressive neuromuscular diseases earlier and reduce the diagnostic gap. Future studies will be needed to better understand differences and similarities in challenges between different patient groups. However, importantly, we did find that patients from younger generations were diagnosed significantly quicker than older generations, indicating a positive trend over the years.

As reported in previous studies in muscle disease, the diagnostic delay depends on the first contacted specialist: A study by Spuler et al. reported a significantly shorter diagnostic delay when patients were seen by specialists compared to when they first presented to general practitioners [25]. In the current study, we found that patients who first presented at an orthopedic specialists received their diagnosis significantly later than patients who were first seen by neurologists, while presenting first symptoms to a general practicioners did not significantly delay diagnosis. Since the first symptoms of CMT often present as orthopedic complications such as foot deformities [2], frequent sprains, or bad performance in school sports, awareness of such factors should be raised to direct orthopedic specialists to think of CMT as a potential underlying cause. Furthermore, neurological misdiagnosis had serious consequences in our study. Previous studies suggest that chronic inflammatory demyelinating polyneuropathy (CIDP) may be the most important misdiagnosis of CMT [26] and related diseases such as transthyrein amyloidosis [27]. Increased awareness across specialties is likely an important step to reduce diagnostic delay in the future. Clinical pathways may be a useful tool to guide diagnostic journeys, and have reduced diagnostic delay in rare (mostly genetic) disease to few months in the example of the National Action League for People with Rare Diseases (TRANSLATE-NAMSE) network in Germany [28].

The psychological burden causing distress and depression in patients with CMT is a relevant aspect of the disease and correlates with disease severity [29]. The way in which and by whom the diagnosis was communicated influenced patients’ distress in our survey. In line with our results, other studies also have shown that when a rare disease diagnosis is communicated, detailed education on the disease and care path is of high relevance [30]. A lack of sufficiently trained healthcare professionals represented a major care gap for patients in our study, consistent with findings from previous research in rare disease [31]. Digital tools may provide a valuable resource in solving the high need for reliable disease information as well as specialist networks and care communities [32].

Apart from psychological and clinical burden for patients due to doubts about their prognosis and not receiving appropriate care, the economic burden of long diagnostic journeys is relevant for patients’ income [33] as well as healthcare systems [34]. More severely affected patients are also less likely to be employed, leaving an important impact on society and indirect cost for family and caregivers [8, 35]. Further prospective studies will be needed to better understand individual care situations and barriers hindering patients to get in touch with specialized care quicker, also regarding regional (including international) similarities and differences. (Digital) tools supporting the patients journey were widely accepted as potential solutions by the patient community in our survey. Such approaches should be developed in respect to scalability to other diseases and regions as well as interoperability with existing systems, and tested for usability in different patient cohorts.

Our study had limitations. While regional and language restrictions limited the survey to Germany, especially Berlin and the surrounding area, a strength of the study lies in the high number of patients reached in a rare disease despite these restrictions. However, patient-reported information bears higher risks to be incomplete, as specific medical knowledge may not be available. This may explain the comparatively low number of patients with the typically most frequent subtype “CMT1A” in this cohort, potentially caused by a high hidden number of CMT1A patients in the anonymous cohort and higher number of rare subtypes in the CMT Registry Study cohort. Furthermore, answers were given subjectively and were potentially modified by recall bias. This especially accounts for participants using the anonymous questionnaire who could not be identified and therefore could not be verified. Future studies may benefit from pseudomomyzed surveys at a larger scale to improve verifiability using medical records.

## Conclusions

This study depicts new insights into the diagnostic odyssey as well as care path of patients with CMT in Germany, revealing diagnostic journeys of more than 10 years and lack of specialized care in Germany. Especially in the light of upcoming new therapy trials and economic impacts of this patient group, new solutions will be needed to address this care gap. As our results replicate previous studies on other rare, and especially inherited neuromuscular disease groups, potential solutions should be scalable to other disease groups.

## Electronic supplementary material

Below is the link to the electronic supplementary material.

Supplementary Material 1

## Data Availability

The datasets used and/or analysed during the current study are available from the corresponding author on reasonable request.

## Abbreviations

CMT: Charcot Marie Tooth disease
CMTR: Charcot–Marie–Tooth and related disorders
DGM: Deutsche Gesellschaft für Muskelkranke GmbH
