# Supplementary material for: Patient journey with Charcot-Marie-Tooth Disease – A German patient survey study
Source: Orphanet J Rare Dis. 2026 Feb 3;21:84. doi: 10.1186/s13023-026-04236-2 (PMC12958679; doi:10.1186/s13023-026-04236-2)
Supplement: Supplementary file 1 — Supplementary Material 1 [file 13023_2026_4236_MOESM1_ESM.docx]

**Supplements**


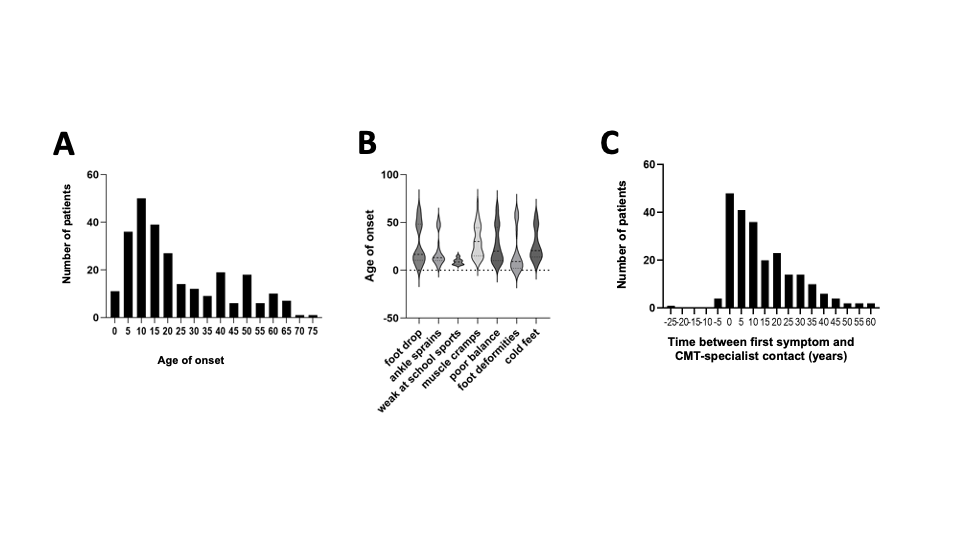


**Suppl. Figure 1.** Disease onset and delay of finding the correct physician. (A) Histogram showing the distribution of ages of onset and (B) violin plots of ages of symptoms depending on specific symptoms., and (C) histogram of time between first clinical contact and specialist visit.

**Suppl. Table 1: Patient Survey (translated from German)**

| **Question** | **Answer Options** |
| --- | --- |
| 1. **General data** | |
| Date | _____________ |
| What is your diagnosis? | ☐ HMSN / CMT (genetic nerve disease)  ☐ ATTRv (genetic amyloidosis – relative answering)  ☐ Muscle disease or other: ___________ |
| Who is completing this survey? | ☐ Patient ☐ Relative (answering for patient) |
| Gender | ☐ Female ☐ Male |
| Current age | Age: _____________ |
| Highest educational degree | ☐ University degree  ☐ Apprenticeship  ☐ School  ☐ None |
| Family origin | ☐ Germany ☐ Portugal ☐ Greece ☐ Ireland ☐ Brazil ☐ Sweden ☐ Japan ☐ Poland ☐ Turkey ☐ Other: ______ |
| Current health status | ☐ Good  ☐ Rather good  ☐ Average  ☐ Rather poor  ☐ Poor |
| 1. **First symptoms and clinical contact before diagnosis** | |
| Age at first symptoms | Age: _____________ |
| Age at first doctor contact | Age: _____________ |
| First doctor seen for symptoms | ☐ GP / Pediatrician  ☐ Orthopedist  ☐ Neurologist  ☐ Cardiologist  ☐ Human geneticist  ☐ Other: ______  ☐ I don’t know |
| Approx. number of doctor visits before correct diagnosis | ☐ 0–1 ☐ 2–5 ☐ 5–10 ☐ >10 ☐ I don’t know |
| Approx. number of different specialists consulted | ☐ 0–1 ☐ 2–5 ☐ 5–10 ☐ >10 ☐ I don’t know |
| Were you hospitalized for diagnosis? | ☐ Never ☐ Once ☐ Several times |
| Contact with specialized center | ☐ Never ☐ At age: ______ ☐ I don’t know |
| How exhausting were the diagnostic tests? | ☐ Not at all ☐ Hardly ☐ Neutral / I don’t know ☐ Rather exhausting ☐ Very exhausting ☐ Not applicable |
| 1. **Diagnosis** | |
| Incorrect diagnoses before final one? | ☐ Never ☐ Once ☐ More than once |
| Incorrect therapies given? | ☐ Yes ☐ No ☐ Not applicable |
| Did misdiagnoses cause long-term problems? | ☐ Yes ☐ No ☐ Not applicable |
| Did misdiagnoses lead to hospital stays? | ☐ Yes ☐ No ☐ Not applicable |
| Age at final diagnosis | Age: _____________ |
| Already knew diagnosis from family history? | ☐ Yes ☐ No |
| Who informed you of the diagnosis? | ☐ GP / Pediatrician  ☐ Orthopedist  ☐ Neurologist  ☐ Cardiologist  ☐ Human geneticist  ☐ Other: ______  ☐ I don’t know |
| How did you feel when you received the diagnosis? | ☐ Very relieved, well cared for  ☐ Rather relieved  ☐ Mixed feelings / I don’t know  ☐ Insecure / lost  ☐ Very afraid, very insecure/lost |
| 1. **Follow-up care** | |
| What happened after diagnosis? (multiple answers possible) | ☐ Discharged, no therapy available  ☐ Immediate follow-up appointment  ☐ Return only if new problems/questions arise  ☐ Participation in studies  ☐ Guidance on slowing disease  ☐ None / I don’t know |
| Frequency of doctor visits since diagnosis | ☐ >2x/year  ☐ 1–2x/year  ☐ Once every few years  ☐ Never  ☐ I don’t know / recent diagnosis  ☐ Not applicable |
| If you have new complaints, who do you contact? | ☐ GP / Pediatrician  ☐ Neurologist  ☐ Specialized center  ☐ Internet  ☐ None  ☐ Not applicable |
| How useful are specialized center visits? | ☐ Very useful  ☐ Quite useful  ☐ Moderate / can’t judge  ☐ Rather not useful  ☐ Not useful |
| Would you use digital connection to a specialized center? | ☐ Yes  ☐ Probably yes  ☐ I don’t know  ☐ Probably no  ☐ No |
| Importance of information & research updates (incl. digital) | ☐ Very important, I’d use digital offers  ☐ Rather important, partial use  ☐ Moderate / can’t judge  ☐ Rather unimportant  ☐ Not important |
| Have you received symptomatic therapy info? (e.g., physiotherapy, aids, pain meds) | ☐ Yes  ☐ No  ☐ Not applicable |
| How helpful are symptomatic therapies? | ☐ Very good, I feel in control  ☐ Good, but some complaints remain  ☐ Partly good, partly bad / can’t judge  ☐ Moderate, little benefit  ☐ Poor, more effort than benefit  ☐ Not applicable |
| Age at first specific therapy (ATTRv only) | ☐ No specific therapy  ☐ At age: ______  ☐ Not applicable |
| Overall, how well do you feel cared for? | ☐ Very well, fully supported  ☐ Rather well, mostly supported  ☐ Neutral / I don’t know  ☐ Rather poorly, sometimes left alone  ☐ Very poorly, many unanswered questions |
| Final comments | Comment: ______________________________________ |
